# Circular RNA Cdr1as sensitizes bladder cancer to cisplatin by upregulating APAF1 expression through miR‐1270 inhibition

**DOI:** 10.1002/1878-0261.12523

**Published:** 2019-06-09

**Authors:** Wenbo Yuan, Rui Zhou, Jingzi Wang, Jie Han, Xiao Yang, Hao Yu, Hongcheng Lu, Xiaolei Zhang, Pengchao Li, Jun Tao, Jifu Wei, Qiang Lu, Haiwei Yang, Min Gu

**Affiliations:** ^1^ Department of Urology The First Affiliated Hospital of Nanjing Medical University China; ^2^ Research Division of Clinical Pharmacology The First Affiliated Hospital of Nanjing Medical University China

**Keywords:** APAF1, bladder cancer, Cdr1as, circular RNA, cisplatin, miR‐1270

## Abstract

Circular RNAs (circRNAs) have recently emerged as essential regulators in carcinogenesis and cancer progression. Previous studies have shown that Cdr1as functions as a microRNA (miRNA) sponge in various cancer types. However, the role of Cdr1as in cisplatin chemosensitivity in bladder cancer remains unclear. Here, we used real‐time PCR to examine miRNA and gene expression in bladder cancer tissues and cell lines. The abilities of Cdr1as and its downstream regulatory molecules to induce apoptosis and promote cisplatin‐induced chemosensitivity of bladder cancer cells were determined by flow cytometry and cell counting kit. Bioinformatic analysis was utilized to predict potential miRNA target sites, and biotin‐coupled miRNA capture, biotin‐coupled probe pull‐down assay, and RNA fluorescent *in situ* hybridization were used to study the interaction between Cdr1as and target miRNAs. Dual‐luciferase reporter assay was also used to validate the target genes of miRNAs. The expression level of apoptotic protease‐activating factor 1 (APAF1) in bladder cancer cells was identified via western blot. Finally, the sensitivity of Cdr1as to cisplatin chemotherapy in nude mice xenografts was evaluated in terms of the size, volume of tumors, and the survival of mice. We report that Cdr1as induced the apoptosis and enhanced the cisplatin chemosensitivity of bladder cancer cells both *in vitro* and *in vivo*. Silencing of APAF1 reduced the sensitivity of bladder cancer cells to cisplatin chemotherapy. Furthermore, Cdr1as could directly sponge miR‐1270 and abolish its effect on APAF1. Our study verified that Cdr1as exerts a cisplatin‐chemosensitization effect on bladder cancer cells through the Cdr1as/miR‐1270/APAF1 axis. This newly identified axis may be a potential therapeutic target for bladder cancer patients.

**Abbreviations**
APAF1: apoptotic protease‐activating factor 1
circRNA: circular RNA
FISH: fluorescence *in situ* hybridization
MIBC: muscle‐invasive bladder cancer
miRNA: microRNA
RIP: RNA immunoprecipitation

## 1 Introduction

Bladder cancer is one of the most common tumors in the human genitourinary system. Approximately 70% of all bladder cancer cases are of the non‐muscle‐invasive type, of which approximately 10–20% cases progress to muscle‐invasive bladder cancer (MIBC; Antoni *et al.*, 2017). MIBC can easily infiltrate other tissues and metastasize; thus, this cancer type is highly lethal (Dobruch *et al.*, 2016). Cisplatin‐based neoadjuvant or adjuvant chemotherapy plays a key role in the comprehensive treatment of MIBC and the main treatment for metastatic bladder cancer (Alfred Witjes *et al.*, 2017). Cisplatin chemotherapy can effectively reduce the recurrence rate of bladder cancer patients, inhibit tumor progression, and improve the prognosis of patients. The therapeutic regimen can increase the 5‐year survival rate of patients with MIBC by up to 58–68% (Meeks *et al.*, 2012). Nevertheless, the overall response rate of cisplatin chemotherapy is only approximately 35% (Sagalowsky, 2013). Cisplatin chemoresistance is the main factor restricting the long‐term survival of bladder cancer patients.

Cisplatin damages cells through a variety of mechanisms, including damage to the cell DNA, intracellular calcium overload, damage to the mitochondria interfering with cell metabolism, activation of the apoptosis pathway, and mediation of the oxidative stress reaction (Chang, 2015). Apoptosis protease‐activating factor 1 (APAF1), as a major factor regulating apoptosis, is a key molecule of the core apoptosis mechanism that forms a ring‐like apoptosome that activates caspase‐3, caspase‐6, or caspase‐7 to execute cell death (Bratton *et al.*, 2001). The free radicals produced by cisplatin can attack the cardiolipin on the mitochondrial membrane, leading to the release of cytochrome C, which, when combined with APAF1, eventually activates the apoptosis pathway and cell death (Hu *et al.*, 1999). Nevertheless, many cancers demonstrate chemoresistance toward cisplatin treatment. Numerous cisplatin‐treated patients must tolerate various side effects without actually benefiting from chemotherapy due to chemoresistance. Interestingly, some studies have demonstrated that APAF1 participates in regulating cisplatin sensitivity (Del Bello *et al.*, 2003; Kamarajan *et al.*, 2001; Kuwahara *et al.*, 2003; Zang *et al.*, 2012). This observation suggests that APAF1 may be a target for regulating the sensitivity of bladder cancer to cisplatin.

Circular RNAs (circRNA) is a covalently closed RNA molecule without a 5′ terminal cap structure and 3′ terminal polyadenylated tail (Jeck *et al.*, 2013). circRNA can function as a competitive endogenous RNA to regulate the biological activity of microRNA (miRNA) and completely or partially restore the inhibitory function of miRNA in the target gene (Memczak *et al.*, 2013). circRNA plays a key role in many biological and pathological processes, including cell proliferation, migration and invasion, metastasis, cell cycle, and apoptosis (Du *et al.*, 2016; Guarnerio *et al.*, 2016; Zhao and Shen, 2017; Zheng *et al.*, 2016). Cdr1as, also known as ciRS‐7 or CDR1NAT, is formed by reverse splicing of the antisense strand of the cerebellar degeneration‐associated antigen 1 (CDR1) gene, which contains 1500 nucleotides (Hansen *et al.*, 2013a, 2013b). It was found that Cdr1as can sponge miR‐7 and regulate downstream target gene expression (Memczak *et al.*, 2013). Other studies have shown that Cdr1as plays oncogenic roles in many tumors, such as hepatocellular carcinoma (Yu *et al.*, 2016) and colorectal cancer (Tang *et al.*, 2017). In our previous study (Li *et al.*, 2018), however, we found that Cdr1as sponges miR‐135a instead of miR‐7, restores p21 activity, and mediates cell cycle arrest in bladder cancer cells. A recent study revealed that circPVT1 mediates docetaxel and cisplatin resistance in osteosarcoma by regulating the drug resistance‐associated gene ABCB1 (Kunpeng *et al.*, 2018). However, the role of Cdr1as in the development of cisplatin chemoresistance in bladder cancer still remains unknown. In the present study, we investigated the relationship between Cdr1as and the sensitivity of bladder cancer to cisplatin and explore the potential miRNA and downstream target gene APAF1 mechanistically. Our research provides novel insights into approaches to reverse cisplatin resistance in bladder cancer and improve the clinical efficacy of the drug.

## 2 Materials and methods

### 2.1 Clinical specimens

Bladder cancer tissues and their paired normal tissues were obtained from patients who were diagnosed with bladder cancer and undergone surgery in the First Affiliated Hospital with Nanjing Medical University (Jiangsu Province Hospital) between 2010 and 2013. The follow‐up deadline was January 2018. All patients signed informed consent before using clinical materials. The use of tissues for this study has been proved by the ethics committee of the First Affiliated Hospital with Nanjing Medical University (Jiangsu Province Hospital). The study methodologies confirmed to the standards set by the Declaration of Helsinki.

### 2.2 Cell culture

Dulbecco’s modified Eagle’s medium containing 10% fetal bovine serum (Gibco, Grand Island, NY, USA) and 1% penicillin–streptomycin were used to culture the human bladder cancer cell lines TCCSUP, 5367, T24, and EJ (Type Culture Collection of the Chinese Academy of Sciences; Shanghai, China) in an incubator with humidified 5% CO_2_ at 37 °C.

### 2.3 Transfection

The Cdr1as plasmid was donated by T. Hansen (Aarhus University, Denmark) and used to construct Cdr1as‐overexpressing adenovirus (Hanbio, Shanghai, China). Cdr1as knockdown lentivirus was also constructed by Hanbio. All miRNA mimics/inhibitors, small‐interfering (si) APAF1, and their controls were synthesized by GenePharma (Shanghai, China) for cell transfection. The transfection procedure was carried out according to the manufacturer’s protocol, and the cells were tested after 48 h of transfection.

### 2.4 IC50 determination

The transfected cells were trypsinized and seeded into a 96‐well plate at a density of 5000 cells per well. Three parallel wells were set up, and the plate was incubated overnight in an incubator. Then, the transfected cells were treated with a series of dilute concentrations of cisplatin (128, 64, 32, 16, 8, 4, 2, and 1 µm, Sigma) for 24 h. Afterward, cell viability was measured by the Cell Counting Kit‐8 (CCK‐8) method (Dojindo, Kumamoto, Japan) according to the manufacturer’s instructions. IC50 was calculated by the probit regression model (Tao *et al.*, 2011). All experiments were independently performed thrice.

### 2.5 Cell apoptosis assays

The transfected cells were stained by an Annexin V‐Alexa Fluor 647/Propidium Iodide Apoptosis Kit (FCmacs, Nanjing, China) and detected by flow cytometry. The ratio of early to late apoptotic cells was counted and compared between the experimental and control groups.

### 2.6 RNA extraction and quantitative real‐time polymerase chain reaction

Total RNAs, including circRNAs and miRNAs, were isolated from tissues and transfected cells by using TRIzol reagent (Invitrogen, Carlsbad, CA, USA) according to the manufacturer’s protocol. cDNA was synthesized using HiScript II (Vazyme, Nanjing, China). Quantitative real‐time polymerase chain reaction (qRT‐PCR) for circRNA and miRNA was performed on an AB7300 thermo‐recycler (Applied Biosystems, Foster City, CA, USA) or LightCycler 480 (Roche, Hillsboro, OR, USA). The primer sequences synthesized by Sangon Biotech (Shanghai, China) were as follows: Cdr1as (forward: 5′‐AGACCTTGAGATTATTGGAAGACTTGA‐3′; reverse: 5′‐TACCCAGTCTTCCATCAACTGGCT‐3′), β‐actin (forward: 5′‐AGCGAGCATCCCCCAAAGTT‐3′; reverse: 5′‐GGGCACGAAGGCTCA TCATT‐3′), GAPDH (forward: 5′‐CGCTCTCTGCTCCTCCTGTTC‐3′; reverse: 5′‐ATCCGTTGACTCCGACCTTCAC‐3′), miR‐1270 mimics (forward: 5′‐CUGGAGAUAUGGAAGAGCUGUGUACAGCUCUUCCAUAUCUCCAGUU‐3′), miR‐1270 inhibitor (forward: 5′‐ACACAGCUCUUCCAUAUCUCCAG‐3′), universal adaptor reverse primer (5′‐GGCCAACCGCGAGAAGATGTTTTTTTTT‐3′), and U6 (forward: 5′‐CTCGCTTCGGCAGCACA‐3′; reverse: 5′‐AACGCTTCACGAATTTGCGT‐3′). β‐Actin, GAPDH, and U6 RNAs were used as internal standard controls for mRNA and miRNA detection, respectively. Each experiment was replicated thrice, and data were analyzed by comparing $2^{-\Delta \Delta C_{\text{T}}}$ values.

### 2.7 Protein extraction and western blot

The transfected cells were lysed with radioimmunoprecipitation assay buffer (Beyotime, Shanghai, China). Total protein was extracted from cell lysates and quantified by BCA (Beyotime). The equivalent amount of protein extract was loaded onto 10% sodium dodecyl sulfate/polyacrylamide gels, separated by electrophoresis, and transferred onto polyvinylidene fluoride membranes (Sigma‐Aldrich, Burlington, MA, USA). The membranes were blocked in 5% nonfat milk in Tris‐buffered saline and Tween‐20 at room temperature for 2 h and then incubated with anti‐APAF1 antibody (1 : 1000; Abcam, Cambridge, UK), anti‐GAPDH (1 : 2000; Cell Signaling Technology, Danvers, MA, USA), or anti‐β‐actin antibody (1 : 2000; Cell Signaling Technology) overnight at 4 °C. Then, the membranes were incubated with a secondary antibody (1 : 5000; Cell Signaling Technology). After washing, the blots were developed with a chemiluminescence system (Bio‐Rad, Hercules, CA, USA) and analyzed by Image Lab Software.

### 2.8 RNA binding protein immunoprecipitation assay

RNA binding protein immunoprecipitation (RIP) assay was conducted using the Magna RIP Kit (Millipore, Danvers, MA, USA) and Ago2 antibody (Cell Signaling Technology) in accordance with the manufacturer’s instructions. The transfected cells were washed with ice‐cold PBS and then mixed with an equivalent volume of RIP lysis buffer. Next, the lysis products were incubated with 5 µg of primary antibodies for 2 h at 4 °C. Subsequently, each sample was mixed with 50 µL of prepared magnetic beads and incubated at 4 °C overnight. The beads were briefly washed (five times in total) with RIP buffer and resuspended in 500 µL of TRIzol LS (Life Technology, Carlsbad, CA, USA). Finally, the RNA of the mixture was extracted and then detected by qRT‐PCR.

### 2.9 Biotin‐coupled miRNA capture

Approximately 3 × 10^6^ cells were transfected with 50 µm of the biotinylated miRNA mimic or nonsense control (NC) (GenePharma, Shanghai, China) for 24 h in an incubator and then lysed in 500 µL of lysis buffer. Subsequently, 50 µL of washed streptavidin magnetic beads (Invitrogen) was blocked for 2 h, added to reaction tubes, and incubated in a rotator (10 r·min^−1^, 4 h, 4 °C) to pull down the biotin‐coupled RNA complex. Subsequently, the beads were briefly washed with lysis buffer (five times in total) and resuspended in 500 µL of TRIzol LS (Life Technology) to recover RNAs specifically interacting with miRNAs. Finally, the binding circRNAs were detected by qRT‐PCR and agarose gel electrophoresis.

### 2.10 Biotin‐coupled probe pull‐down assay

The specific biotinylated probes designed to bind to the junction area of Cdr1as were synthesized by Gongsi, China, whereas the oligo probe was taken as the control. The sequences were as follows: Cdr1as sense: 5′‐GGTGCCATCGGAAACCCTGGATATTGCAGACA‐3′‐Biotin and Oligo: 5′‐TGTCTGCAATATCCAGGGTTTCCGATGGCACC‐3′‐Biotin. The transfected cells (approximately 1 × 10^7^) were washed with ice‐cold PBS, lysed in 500 µL of lysis buffer, and then incubated with 3 μg biotinylated probes at room temperature for 2 h. Subsequently, 50 µL of blocked streptavidin magnetic beads (Invitrogen) was added to the biotin‐coupled RNA complex, which was incubated for another 4 h at 4 °C. The beads were briefly washed with lysis buffer (five times in total), and the binding miRNAs in the pull‐down products were extracted using TRIzol LS (Life Technology) and detected using qRT‐PCR.

### 2.11 Agarose gel electrophoresis

Exactly 0.8 g of agarose powder was weighed in a conical bottle. Then, 40 mL of 0.5× TAE solution was weighed in a measuring cylinder and poured into the same conical bottle. The conical bottle was placed into an induction cooker to heat for 3–5 min. The agarose gel was completely boiled, and 1 µm nucleic acid dye was added to it. The liquid agarose gel was quickly poured into the mold, inserted with a 10‐hole comb, and solidified for 30 min at room temperature. The gel was placed into the electrophoresis tank, and 0.5× TAE solution was poured over it until the gel was just immersed. About 5 µL of the PCR product was mixed with 1 µL of the DNA ladder, added to the agarose gel hole, and subjected to constant‐pressure 90 V electrophoresis for 20–30 min. After electrophoresis, the gel was placed under an ultraviolet lamp to observe the position and grayscale of the DNA strip.

### 2.12 Fluorescence *in situ* hybridization

DNA oligo probes (GenePharma) labeled with Cy5 for Cdr1as (5′‐GGTGCCATCGGAAACCCTGGATATTGCAGACA‐3′‐Cy5) and FAM for miR‐1270 (5′‐ACACAGCTCTTCCATATCTCCAG‐3′‐FAM) were used in the fluorescence *in situ* hybridization (FISH) assays, wherein the nuclei were counterstained with 4,6‐diamidino‐2‐phenylindole. All procedures were performed in accordance with the manufacturer’s instructions (GenePharma), and all images were acquired using a Leica SP5 confocal microscope (Leica Microsystems, Mannheim, Germany).

### 2.13 Luciferase reporter assay

T24 cells were cultured in 24‐well plates and then cotransfected with plasmid containing psiCHECK‐APAF1‐wt or psiCHECK‐APAF1‐mut together with Firefly and Renilla luciferase and miR‐1270 mimics or the control by using Lipofectamine 3000 (Invitrogen) in accordance with the manufacturer’s protocol. After transfection for 24 h, Firefly and Renilla luciferase activities were measured using the dual‐luciferase reporter assay system (Promega, Madison, WI, USA). The ratios of luminescence from Firefly to Renilla luciferase were normalized through three independent experiments.

### 2.14 Xenografts in mice

The animal studies were approved by the Animal Management Committee of Nanjing Medical University. T24 cells were transfected with the Cdr1as adenovirus and GFP vector. Female athymic BALB/c nude mice (4 weeks old, *n* = 20) were used. The nude mice were randomly divided into four groups, namely the Cdr1as + cisplatin group, the CTL + cisplatin group, the Cdr1as + saline group, and the CTL + saline group, and five rats were assigned to each group. Approximately 10^7^‐transfected T24 cells and control cells were injected subcutaneously into the axilla of the mice. The width (W) and length (L) of the developed tumors were measured using calipers every 3 days, and tumor volume (V) was calculated using the formula V = (W^2^ × L)/ 2. A week after injection, cisplatin (10 mg·kg^−1^) was intraperitoneally injected into mice in the experimental groups every 3 days, while mice in the control group were injected with the same amount of normal saline. At 3 week after injection, the mice were sacrificed, and tumor weights were measured. The tumors were fixed in 4% formalin for immunohistochemical analysis. In mouse survival assay, 4‐week‐old nude mice were randomly divided into four groups, namely as before, and each group had ten mice. All mice were injected with T24 cells (6 × 10^6^ cells/mouse) through tail vein and two weeks later injected with saline (with or without cisplatin 10 mg·kg^−1^) intraperitoneally every 3 days for 3 weeks. Mouse survival was monitored throughout the assay, during which *in vivo* tumor growth and metastases were partly detected with the IVIS 100 Imaging System (Xenogen, Los Angeles, CA, USA).

### 2.15 Tissue microarray and immunohistochemistry

Tissue microarray (TMA) was constructed using 160 cases of formalin‐fixed, paraffin‐embedded bladder cancer samples. Immunohistochemistry (IHC) was performed on TMA to evaluate APAF1 protein expression, also on paraffin‐embedded tissues from nude mice. Tissue sections and TMAs were dewaxed and then rehydrated in graded ethanol. Tissue sections were placed in sodium citrate buffer (pH = 6), and the antigen was extracted by microwave heating. Tissue sections were soaked in 3% H_2_O_2_ for 10 min and then incubated with APAF1 antibody (Abcam Company, 1 : 400) at 4 °C overnight. After washing, the tissue sections were incubated with horseradish peroxidase‐conjugated rabbit antibody for 30 min at room temperature. After washing once more, the tissue sections were stained with fresh 3,3′‐diaminobenzidine, and images were obtained under a microscope (Olympus, Tokyo, Japan) with the appropriate magnification.

Stained tissues were scored for staining intensity (SI) and the percentage of positive cells (PP). SI was scored on a scale of 0–3 (0, negative staining; 1, weak staining; 2, moderate staining; and 3, strong staining), and PP was scored into five categories: 0 (0% PP), 1 (< 10%), 2 (11–50%), 3 (51–80%), or 4 (> 80%). The final staining score was calculated by multiplying SI and PP score, resulting in a score value ranging from 0 to 12. The positive level of immunohistochemical staining was scored by two urologists, and patients with different scores were divided into low‐ (0–6) and high‐staining (7–12) groups.

### 2.16 Bioinformatics analysis

We downloaded the bladder cancer patient data from the TCGA data collection (https://xenabrowser.net/datapages/). The database is generated by the TCGA Research Network: http://cancergenome.nih.gov/. GO pathway analysis was obtained from http://geneontology.org/. Gene expression and survival data were got from http://ualcan.path.uab.edu/. Median expression of APAF1 and miR‐1270 in cancer tissues was used as a cutoff value in survival analysis. Original data were acquired from TCGA.

### 2.17 Statistical analysis

Data were analyzed using spss version 22.0 (IBM, Armonk, NY, USA) and are presented as mean ± standard deviation. Two‐tailed Student’s *t*‐test and one‐way ANOVA were performed to analyze differences between the groups. Correlations were analyzed by Pearson’s correlation method and Spearman’s rank correlation test. Survival curves were plotted using the Kaplan–Meier method, and differences were analyzed by the log‐rank test. In all statistical tests, *P* < 0.05 was considered statistically significant.

## 3 Results

### 3.1 Cdr1as enhances the cisplatin chemosensitivity of bladder cancer cells *in vitro* and *in vivo*

**Figure 1:**
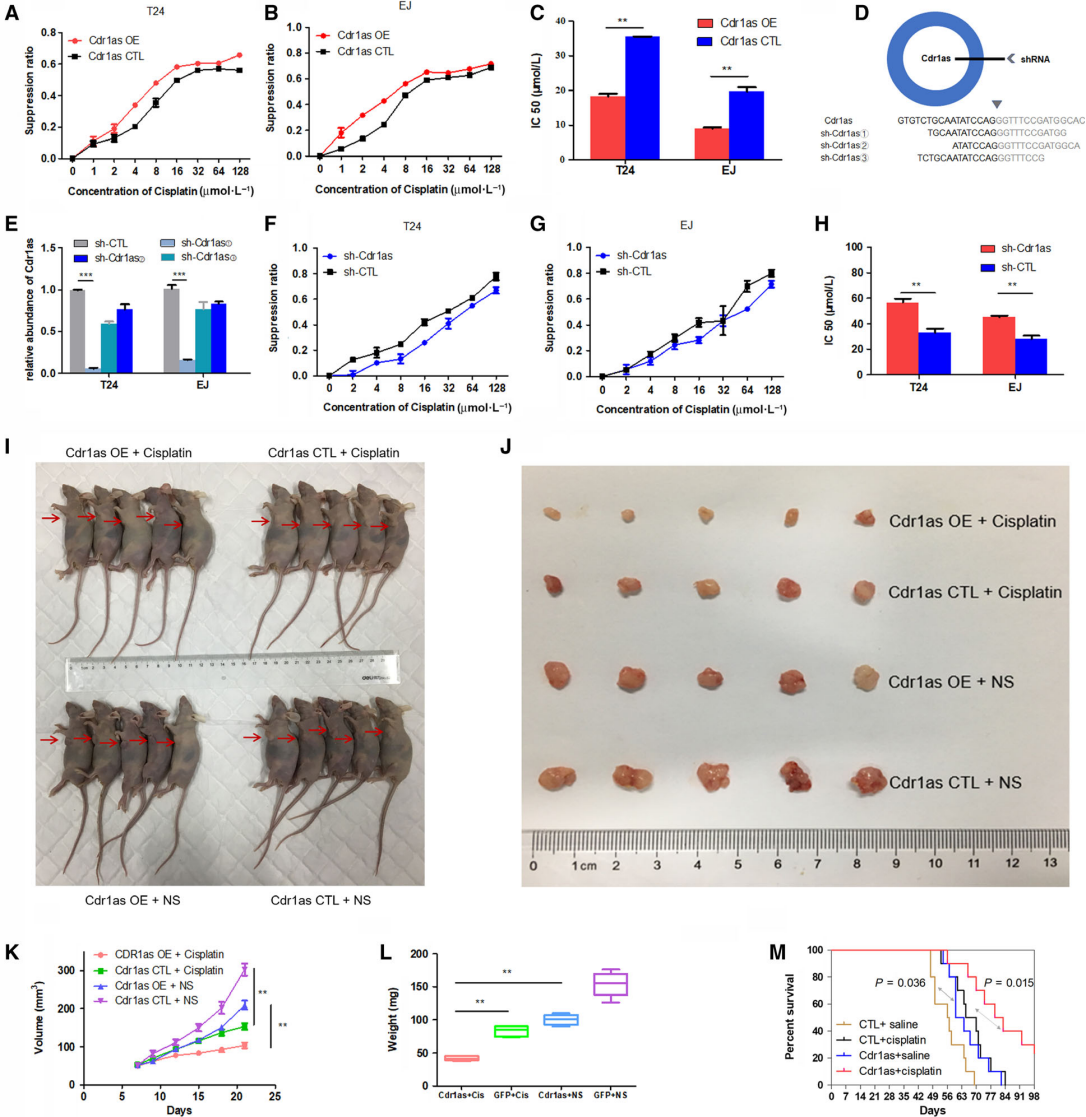
Cdr1as enhances the cisplatin chemosensitivity *in vitro* and *in vivo*. (A, B) CCK‐8 assay showed that overexpression of Cdr1as could dramatically inhibit the proliferation of T24 and EJ cells in a series of dilute concentrations of cisplatin. (C) Transfection of Cdr1as OE (overexpressing) adenovirus increased the sensitivity of T24 and EJ cells to cisplatin. (D) The sketch of structure of sh‐Cdr1as vectors was shown. (E) The expression levels of Cdr1as were performed by using qRT‐PCR in T24 and EJ cells transfected with three kinds of sh‐Cdr1as or sh‐CTL (control) vector, respectively. We used sh‐Cdr1as① to conduct Cdr1as‐downregulated‐associated assays with sh‐Cdr1as② and sh‐Cdr1as③ eliminated. (F, G) Cells viability was determined by the CCK‐8 method to be promoted by sh‐Cdr1as‐transfected cells in a series of dilute concentrations of cisplatin. (H) Knocking down Cdr1as could reduce the sensitivity of T24 and EJ cells to cisplatin. (I) Axillary tumor formation in nude mice. (J) Collation map of tumor specimens among four groups. (K) Volume of xenografted tumor was measured using vernier calipers every week. (L) Summary of xenografted tumor weights of mice in four groups which were measured using electronic scales. (M) Nude mice survival assay showed Cdr1as‐overexpressed bladder tumors are more sensitive to cisplatin *in vivo*. The Cdr1as + cisplatin group lives longer than the Cdr1as + saline and much longer than the control + saline group. Data represent the mean ± SD from three independent experiments. Student’s *t*‐test with two biological independent replicates and log‐rank test were used to determine statistical significance; **P < 0.01, ***P < 0.001.

To investigate the effects of Cdr1as on bladder cancer cells, we infected T24 and EJ cells with the Cdr1as‐overexpressing adenovirus (Cdr1as OE) or control GFP adenovirus (Cdr1as CTL). qRT‐PCR assay indicated the relative abundance of Cdr1as in all available bladder cancer cell lines (Fig. S1A,B) and in cells infected with adenovirus (Fig. S1C). CCK‐8 assay showed that Cdr1as overexpression could dramatically inhibit the proliferation of T24 and EJ cells in a series of dilute concentrations of cisplatin (Fig. 1A,B). The IC50 of cisplatin to T24 and EJ cells in the Cdr1as OE group was significantly lower than that in the Cdr1as CTL group (Fig. 1C). To confirm the effect of Cdr1as on the chemosensitivity of bladder cancer cells to cisplatin, we also constructed three kinds of Cdr1as knockdown lentivirus (sh‐Cdr1as①, ②, and ③, Fig. 1D) and GFP lentivirus (sh‐CTL). qRT‐PCR confirmed that Cdr1as expression level was significantly downregulated in T24 and EJ cells by sh‐Cdr1as① instead of sh‐Cdr1as② and sh‐Cdr1as③ (Figs 1E and Fig S1D), and the opposite phenomenon was observed (Fig. 1F‐H). These results demonstrate that Cdr1as enhances the cisplatin chemosensitivity of bladder cancer cells.

We also knocked down the expression of Cdr1as in TCCSUP and 5637 cells by the Cdr1as knockdown lentivirus. qRT‐PCR confirmed that Cdr1as expression level was significantly downregulated in TCCSUP by sh‐Cdr1as① and sh‐Cdr1as②, which in 5637 cells by sh‐Cdr1as① (Fig. S2A). CCK‐8 assay showed that Cdr1as knockdown could promote proliferation of TCCSUP and 5367 cells in a series of dilute concentrations of cisplatin (Fig. S2B,C). The IC50 of cisplatin in the sh‐Cdr1as group was higher than that in the sh‐NC group (Fig. S2D). These results in TCCSUP and 5637 cells were consistent with the results in T24 and EJ cells.

T24 cells transfected with Cdr1as or GFP were injected subcutaneously into nude mice to investigate whether Cdr1as overexpression affects the cisplatin chemosensitivity of tumors *in vivo*. Tumor volumes were measured weekly after injection (Fig. 1I,J), and the volume and quality of xenografts in the cisplatin chemotherapy group were lower than those in the normal saline control group (Fig. 1K,L). Compared with those of the Cdr1as CTL group, the tumor volume and quality of the Cdr1as‐overexpressing group were significantly reduced after cisplatin chemotherapy (Fig. 1K,L).

In mouse survival assay, mice were injected with T24 cells through tail vein. No signal was detected in a normal mouse by the IVIS 100 Imaging System, and its normal lungs are shown in Fig. S3A. Signals of metastasis in a mouse’s body were detected by the IVIS 100 Imaging System, and metastasis in its lungs was observed after its death (Fig. S3B). The results showed that mice in the Cdr1as + saline group lived longer than those in the CTL + saline group, while mice in the Cdr1as + cisplatin group lived longer than those in the CTL + cisplatin group (Fig. 1M).

### 3.2 Cdr1as induces cell apoptosis in bladder cancer cells

**Figure 2:**
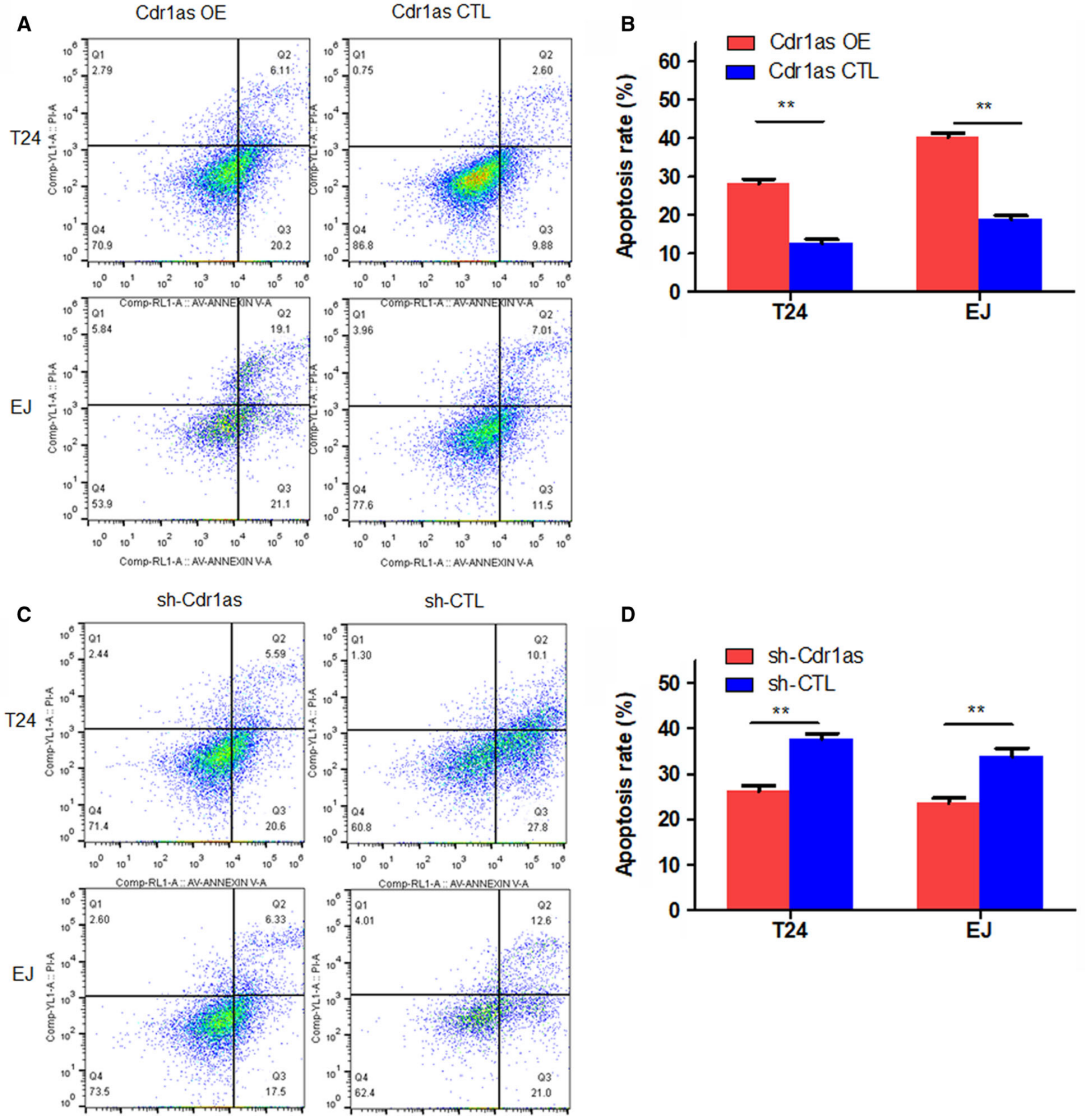
Cdr1as induces cell apoptosis in bladder cancer cells. (A, B) Overexpression of Cdr1as in T24 and EJ cells promoted cell apoptosis using Annexin V‐FITC/PI staining by the flow cytometry. (C, D) Knocking down Cdr1as in T24 and EJ cells increased cell viability. Data represent the mean ± SD from three independent experiments. Student’s *t*‐test with two biological independent replicates was used to determine statistical significance; **P < 0.01.

Flow cytometry was performed to detect the effect of Cdr1as on cell apoptosis. Compared with that of the Cdr1as CTL group, the apoptotic rate (early apoptosis + late apoptosis) of T24 and EJ cells increased significantly after Cdr1as overexpression (Fig. 2A,B); the opposite trend was observed in the sh‐Cdr1as and sh‐CTL groups (Fig. 2C,D). The same results were also observed in the knockdown Cdr1as of TCCSUP and 5637 cells (Fig. S4A,B). These results indicate that Cdr1as induces cell apoptosis in bladder cancer cells.

### 3.3 Cdr1as acts as a molecular sponge for miR‐1270

**Figure 3:**
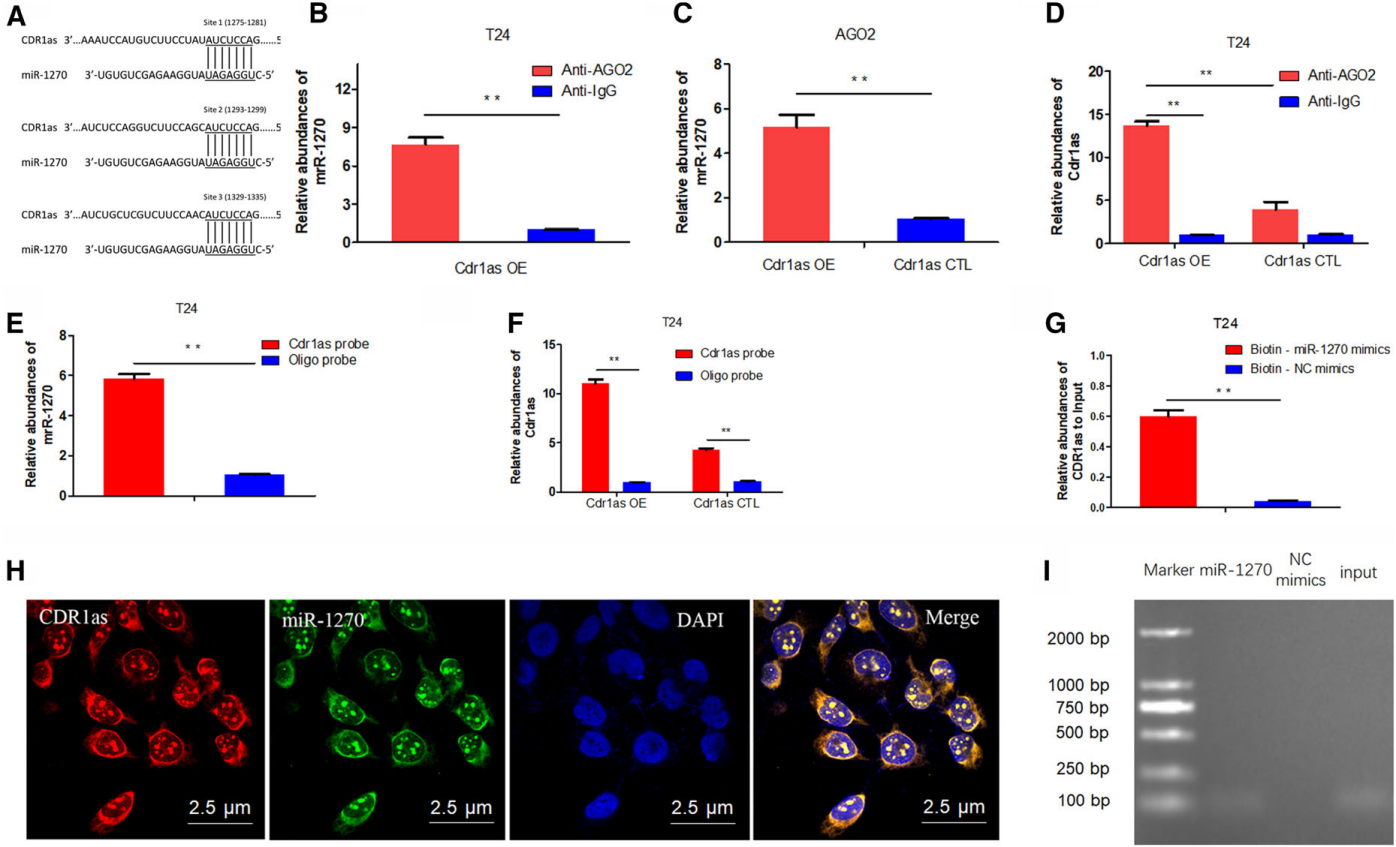
Cdr1as acts as a molecular sponge for miR‐1270. (A) Three putative miR‐1270 binding sites on Cdr1as. (B) The amount of Cdr1as in Cdr1as OE or Cdr1as CTL groups was detected by Ago2 RIP assay. (C) Relative enrichment level of miR‐1270 immunoprecipitated by Ago2 in Cdr1as OE or Cdr1as CTL groups. (D) Relative enrichment level of miR‐1270 in Cdr1as OE group immunoprecipitated by Ago2 or IgG. (E) Relative enrichment level of miR‐1270 pulled down by biotinylated Cdr1as probes or Oligo probes in Cdr1as OE group. (F) Relative enrichment level of Cdr1as pulled down by biotinylated Cdr1as probes or Oligo probes in Cdr1as OE or Cdr1as CTL groups, respectively. (G) Relative enrichment level of Cdr1as pulled down by biotinylated miR‐1270 mimics or nonsense control (NC) mimics in Cdr1as OE group. (H) Cdr1as and miR‐1270 were colocalized in T24 cell by FISH. Cdr1as was stained red, miR‐1270 was stained green, and nuclei were stained blue (DAPI) (Scale bar, 2.5 μm). (I) Cdr1as in pull‐down products from G was amplified by PCR and then detected by agarose gel electrophoresis. Data represent the mean ± SD from three independent experiments. Student’s *t*‐test with two biological independent replicates was used to determine statistical significance; **P < 0.01.

Circular RNAs function mainly as miRNA sponges to bind functional miRNAs and then regulate gene expression (Hansen *et al.*, 2013a, 2013bb). Our previous study preliminarily demonstrated that miR‐1246, miR‐7, and miR‐135a could bind to Cdr1as. We further found that miR‐1270 could also bind to Cdr1as and three binding sites are shown as illustrated (Fig. 3A). AGO2 RIP assay showed that AGO2 adsorbs more Cdr1as than IG (over 10‐fold) in Cdr1as‐transfected cells (Fig. 3B). The miR‐1270 levels pulled down by Ago2 in Cdr1as OE cells were approximately fivefold greater than those in Cdr1as CTL‐transfected cells (Fig. 3C). In Cdr1as OE cells, more miR‐1270 was observed in the Ago2 pull‐down eluents than in the IG pull‐down products (Fig. 3D). We then applied the biotin‐coupled probe pull‐down assay to further confirm this interaction. As shown in Fig. 3E,F, specific enrichment of Cdr1as and miR‐1270 was detected in the Cdr1as pulled down pellet compared with that in the Oligo group. This result indicates that Cdr1as could directly sponge miR‐1270. We conducted biotin‐coupled miRNA capture and FISH to confirm the sponge effect of Cdr1as. Biotin‐coupled miR‐1270 analogously captured more Cdr1as than biotin‐coupled NC. This phenomenon indicates that miR‐1270 could bind to Cdr1as (Fig. 3G). PCR experiments were conducted to find out the relative abundance of miR‐1270 in tumors of mice formed by T24 cells infected with Cdr1as or control. And we found less miR‐1270 in Cdr1as‐overexpressed tumors (Fig. 3H). FISH technology showed that Cdr1as (red fluorescence) and miR‐1270 (green fluorescence) could be visualized in the cells, and colocalization was observed in T24 cells (Fig. 3I). Finally, the Cdr1as expression in the RNA pull‐down products was verified by agarose gel (Fig. 3J). Taken together, the results indicate that Cdr1as could serve as a sponge for miR‐1270 in bladder cancer.

### 3.4 miR‐1270 plays an oncogenic role in bladder cancer cells and reduces their cisplatin chemosensitivity

**Figure 4:**
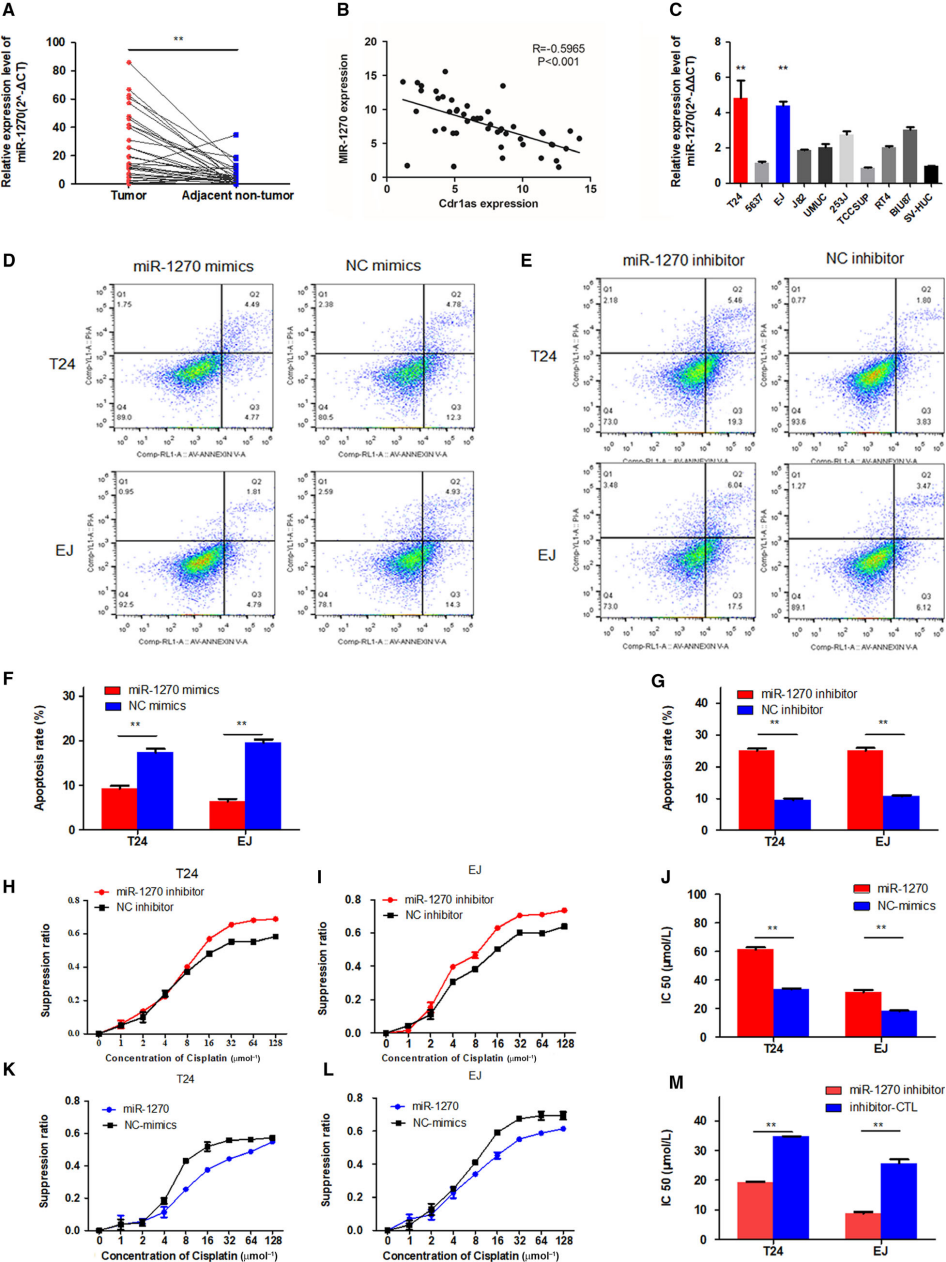
miR‐1270 plays an oncogenic role in bladder cancer cells and reduces cisplatin chemosensitivity. (A) Relative expression level of miR‐1270 in bladder cancer tissues and matched adjacent normal tissues (*n* = 32) using qRT‐PCR. miR‐1270 was significantly upregulated in bladder cancer tissues. (B) The expression of Cdr1as and miR‐1270 in bladder cancer samples had a moderate negative correlation confirmed by Pearson’s correlation analysis. (C) Relative expression level of miR‐1270 in bladder cancer cell lines and SV‐HUC‐1 cell using qRT‐PCR. (D) Overexpression of miR‐1270 in T24 and EJ cells decreased cell apoptosis using Annexin V‐FITC/PI staining by the flow cytometry. (E) Downregulated miR‐1270 strengthened cell apoptosis using Annexin V‐FITC/PI staining by the flow cytometry. (F, G) Transfection of miR‐1270 mimics decreased the sensitivity of T24 and EJ cells to cisplatin. Meanwhile, the opposite phenomenon was observed in miR‐1270‐downregulated T24 and EJ cells. (H, I, K, L) Cell viability was determined by the CCK‐8 method to be inhibited by miR‐1270 mimics or inhibitor‐transfected cells in a series of dilute concentrations of cisplatin. (J, M) Overexpression of miR‐1270 could decrease the sensitivity of T24 and EJ cells to cisplatin, while the weakening the expression of miR‐1270 could increase the sensitivity of T24 and EJ cells to cisplatin. Data represent the mean ± SD from three independent experiments. Student’s *t*‐test with two biological dependent or independent replicates was used to determine statistical significance; **P < 0.01.

To investigate whether miR‐1270 expression is altered in bladder cancer, we examined the miR‐1270 expression in 32 pairs of bladder cancer tissues and matched adjacent normal tissues via qRT‐PCR. The clinicopathological characteristics of the samples are presented in Table S1. As shown in Fig. 4A, the expression level of miR‐1270 in bladder cancer tissues was significantly increased compared with that in adjacent nontumor tissues. Correlation analysis demonstrated that the expression of Cdr1as showed a moderately negative correlation with miR‐1270 (*R* = −0.5965, *P* < 0.001, Fig. 4B). We also confirmed that miR‐1270 showed higher expression in seven BCA cell lines (T24, EJ, J82, UMUC, 253J, RT4, and BIU‐87) compared with that in SV‐HUC‐1, which is considered the normal urothelial cell line (Fig. 4C). Compared with that in the NC mimic group, the apoptosis rate of T24 and EJ cells decreased significantly after miR‐1270 overexpression (Fig. 4D,F). The opposite trend was observed when the miR‐1270 and NC inhibitor groups were compared (Fig. 4E,G).

We explored the effects of four potential miRNAs (miR‐1246, miR‐7, miR‐135a, and miR‐1270) on the cisplatin chemosensitivity of bladder cancer cells and found that only miR‐1270 induced significant changes in this chemosensitivity (data of other miRNAs are shown in Fig. S5A–D). As shown in Fig. 4H,I, the cell growth inhibition rate of the miR‐1270 overexpression group was significantly lower than that of the control group. The probit regression model showed that the IC50 of cisplatin in the miR‐1270 overexpression group was higher than that in the control group (Fig. 4J). These results could be reversed after knocking down miR‐1270 (Fig. 4K–M). Taken together, the findings thus far indicate that miR‐1270 plays an oncogenic role in bladder cancer cells and reduces their cisplatin chemosensitivity.

### 3.5 miR‐1270 can partially reverse the effect of Cdr1as on cisplatin chemosensitivity and cell apoptosis in bladder cancer cells

**Figure 5:**
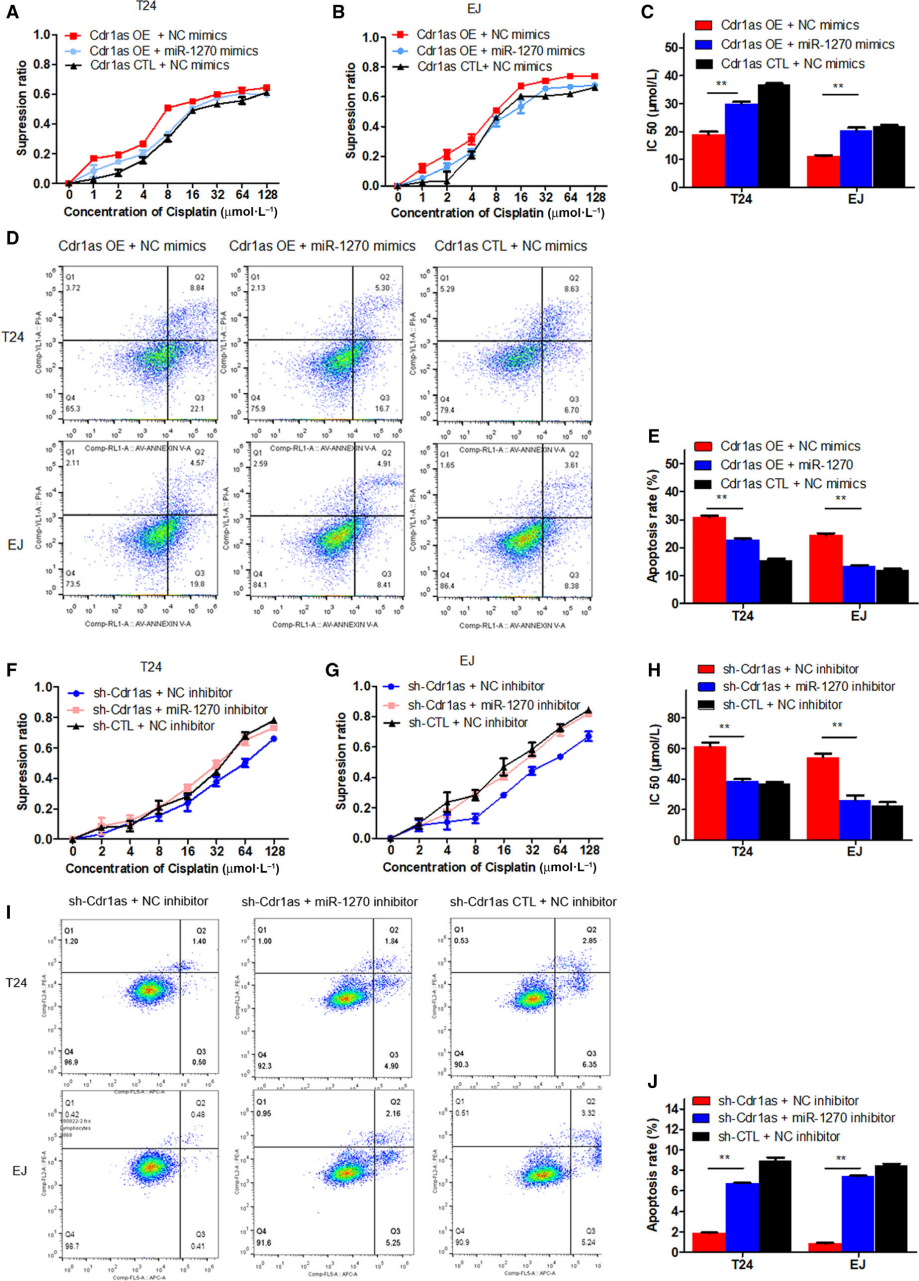
miR‐1270 could partially reverse the effect of Cdr1as on cell apoptosis in bladder cancer cells. (A, B) CCK‐8 assay showed that overexpression of miR‐1270 partially restored the cell proliferation in a series of dilute concentrations of cisplatin induced by cotransfection of Cdr1as OE and miR‐1270 mimics in T24 and EJ cells. (C) Upregulation of miR‐1270 partially reversed the chemosensitivity induced by overexpressing of Cdr1as in T24 and EJ cells. (D, E) Upregulation of miR‐1270 partially reversed the increment of T24 and EJ cells apoptosis induced by overexpression of Cdr1as in T24 and EJ cells. (F, G) CCK‐8 assay showed that weakening the expression of miR‐1270 partially reversed the cell proliferation in a series of dilute concentrations of cisplatin induced by cotransfection of sh‐Cdr1as and miR‐1270 inhibitor in T24 and EJ cells. (H) Downregulation of miR‐1270 partially restored the chemosensitivity induced by knocking down Cdr1as in T24 and EJ cells. (I, J) Downregulation of miR‐1270 partially reversed the reduction of T24 and EJ cell apoptosis induced by knocking down Cdr1as in T24 and EJ cells. Data represent the mean ± SD from three independent experiments. Student’s *t*‐test with two biological independent replicates was used to determine statistical significance; **P < 0.01.

We first constructed T24 and EJ cells cotransfected with the Cdr1as adenovirus and miR‐1270 mimics to further explore the functional relationship between Cdr1as and miR‐1270. The results showed that miR‐1270 mimics could partially attenuate the Cdr1as overexpression‐mediated inhibition of cisplatin chemosensitivity in bladder cancer cells (Fig. 5A–C). Moreover, cotransfection of Cdr1as and miR‐1270 partially reversed the apoptosis induced by overexpressing Cdr1as (Fig. 5D,E).

We revalidated this result by building T24 and EJ cells cotransfected with the sh‐Cdr1as lentivirus and miR‐1270 inhibitor. After knocking down Cdr1as and miR‐1270 simultaneously, the cisplatin chemosensitivity induced by sh‐Cdr1as alone was partially reversed in T24 and EJ cells (Fig. 5F‐H). Similarly, the decrease in T24 and EJ cell apoptosis induced by knocking down Cdr1as was partially reversed by cotransfection of the sh‐Cdr1as lentivirus and miR‐1270 inhibitor (Fig. 5I,J). Thus, miR‐1270 can partially reverse the effect of Cdr1as on the cisplatin chemosensitivity and apoptosis of bladder cancer cells.

### 3.6 APAF1 is the direct target gene of miR‐1270, which could enhance the cisplatin chemosensitivity of bladder cancer cells and induce their apoptosis

**Figure 6:**
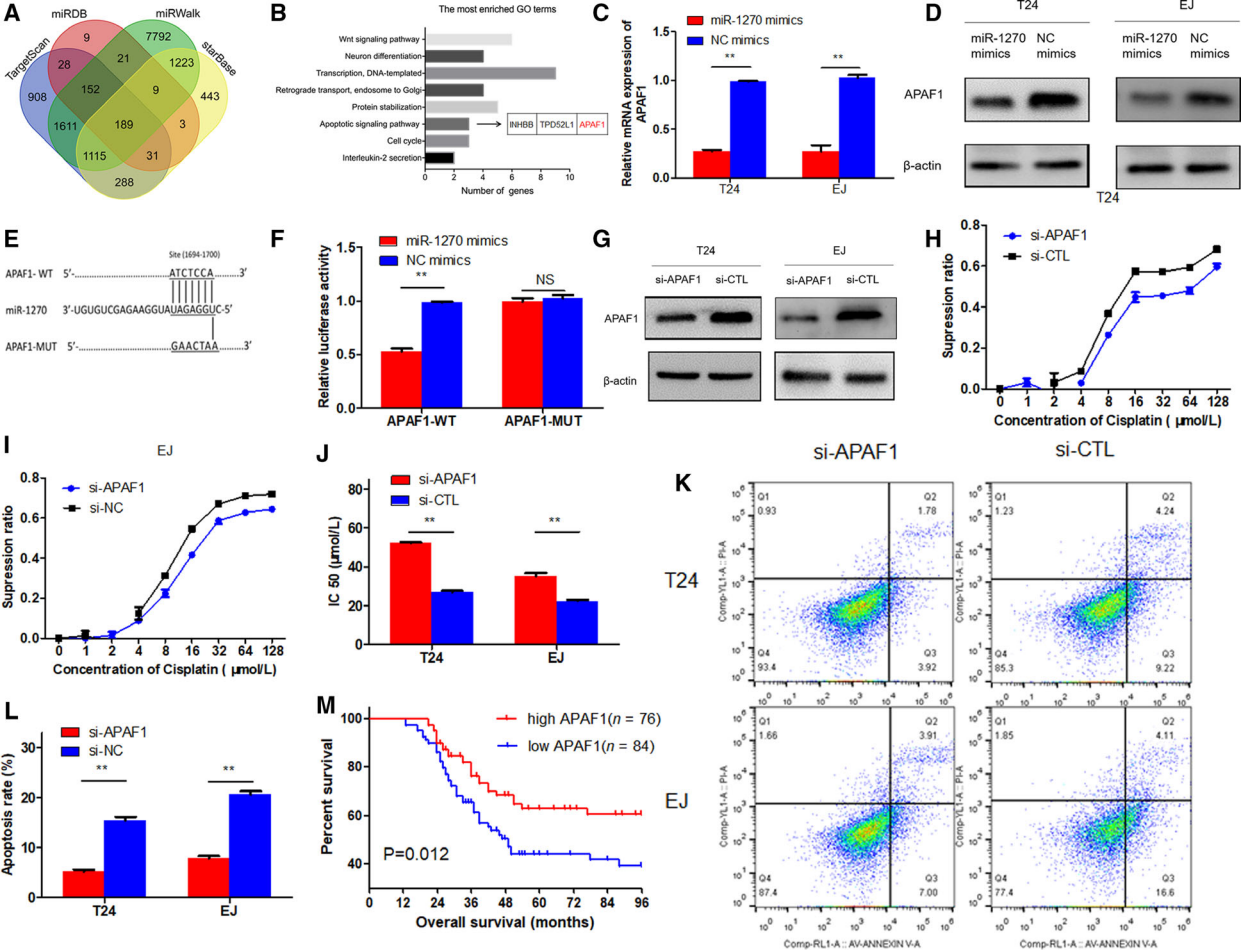
Cdr1as modulated the expression of endogenous miR‐1270 targets APAF1. (A) Venn diagram representing the overlap of target genes of miR‐1270 based on four algorithms (TargetScan, MIRDB, miRWalk, and starBase). The Venn diagram tool is available at http://bioinformatics.psb.ugent.be/webtools/Venn/. (B) Gene enrichment analysis for all the common 189 targets. (C, D) miR‐1270 inhibited the mRNA and protein expression level of APAF1. (E) Potential miR‐1270 binding sequence in the 3’‐UTR of APAF1 mRNA. (F) APAF1 was the direct target of miR‐1270 through dual‐luciferase reporter assays. (G) Western blot confirmed that APAF1 was knocked down in T24 and EJ cells. (H, I) Cells viability was determined by the CCK‐8 method to be promoted by si‐APAF1‐transfected cells in a series of dilute concentrations of cisplatin. (J) Knocking down APAF1 could reduce the sensitivity of T24 and EJ cells to cisplatin. (K, L) Knocking down APAF1 in T24 and EJ cells decreased cell apoptosis. (M) Kaplan–Meier survival analysis for OS of patients in TMAs according to APAF1 expression status. Data represent the mean ± SD from three independent experiments. Student’s *t*‐test with two biological independent replicates and log‐rank test were used to determine statistical significance; **P < 0.01.

We found 189 miR‐1270‐related potential target genes through 4 database [starBase, miRWalk, miRDB, and TargetScan (TS)] (Fig. 6A). All targets of miR‐1270 are listed in the Tables S2–S3. Then, we did GO analysis and found apoptotic signaling pathway was one of the eight pathways. The apoptotic signaling pathway contained three genes INHBB, TPD52L1, and APAF1 (Fig. 6B). We chose APAF1 to continue our research because it was considered as the most correlated with tumors and chemotherapy. As shown in Fig. 6C,D, miR‐1270 overexpression could decrease the mRNA level of APAF1 and decrease its protein expression levels. We performed dual‐luciferase reporter assay and found that cotransfection of miR‐1270 mimics and reporter plasmids strongly reduced luciferase activity. Conversely, cotransfection of miR‐1270 mimics and mutated vectors showed no obvious effect on the luciferase activity (Fig. 6E,F). Consequently, the results prove that APAF1 is the direct target gene of miR‐1270.

To investigate the effects of APAF1 on bladder cancer cells, we transfected si APAF1 or si‐CTL into T24 and EJ cells and subsequently detected protein levels of APAF1 by western blot. The results showed depressed APAF1 expression (Fig. 6G). Compared with those in the si‐CTL group, cells transfected with si‐APAF1 revealed significantly increased viability and less sensitivity to cisplatin in a series of dilute concentrations (Fig. 6H–J). In addition, APAF1 blockage also markedly inhibited cell apoptosis in T24 and EJ cells (Fig. 6K,L). The same results were found in cells overexpressing miR‐1270, which suggests that miR‐1270 regulates cisplatin chemosensitivity in bladder cancer by targeting APAF1. Furthermore, we investigated the expression of APAF1 in TMA. APAF1 was expressed in the nucleus and the cytoplasm (Fig. S6). The result showed that the expression of APAF1 was related to tumor histological grade by IHC analysis in TMA. The histological grade was higher in the high expression of APAF1 group (Table 1). Finally, Kaplan–Meier survival curves showed patients with low APAF1 expression had worse prognosis and shorter survival time, compared to those with high APAF1 expression (Fig. 6M), which was consistent with the results from tumor samples with detailed clinical information which was downloaded from TCGA database (APAF1 low *n* = 197, APAF1 high *n* = 198, *P* = 0.0101, Fig. S7A). But no significant difference of overall survival was observed according to miR‐1270 level in bladder tumor samples with detailed clinical information which was downloaded from TCGA database (miR‐1270 low *n* = 197, miR‐1270 high *n* = 198, *P* = 0.3089, Fig. S7B).

**Table 1:** Relationship between APAF1 expression and clinicopathological features of bladder cancer patients.

Variable	Number of cases	APAF1 expression		*P*‐value
		Low	High	
Cases	160	84	76	
Age at surgery (years)				
≥ 68	83	47	36	0.056
< 68	77	37	40	
Gender				
Male	125	64	61	0.302
Female	35	20	15	
Pathological stage				
pTa–pT1	29	13	16	0.075
pT2–pT4	131	71	60	
Tumor grade				
Low	59	27	32	0.014*
High	101	57	44	
Tumor size(cm)				
< 3	92	47	45	0.133
≥ 3	68	37	31	

* *P* < 0.05 is considered significant.

### 3.7 Cdr1as plays a regulatory role through the Cdr1as/miR‐1270/APAF1 axis

**Figure 7:**
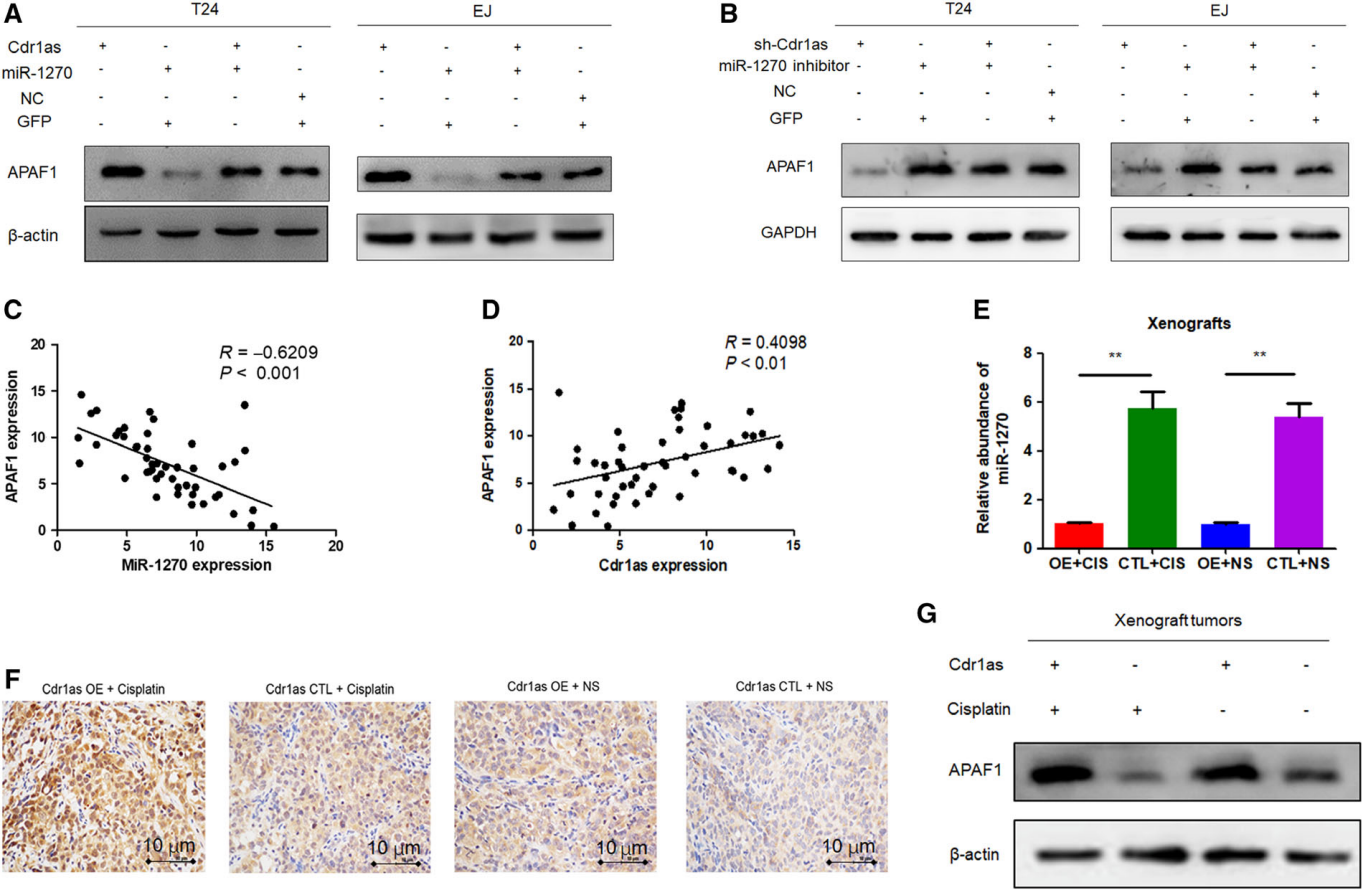
Cdr1as plays a regulatory role through the Cdr1as/miR‐1270/APAF1 axis. (A) Western blot showed that miR‐1270 could partly decrease the protein expression level of APAF1 in Cdr1as OE cells. (B) Western blot showed that miR inhibitor could partly increase the protein expression level of APAF1 in sh‐Cdr1as cells. (C) Negative correlation between the expression level of Cdr1as and miR‐1270 in bladder cancer samples. (D) Moderate positive correlation between the expression level of Cdr1as and APAF1 in bladder cancer samples. (E) miR‐1270 abundance in xenografts. (F) APAF1 IHC staining of xenografted tumors retrieved from treated nude mice (200×) (Scale bar, 10 μm). (G) APAF1 western blot of tumors in the four groups. Data represent the mean ± SD from three independent experiments. Student’s *t*‐test with two biological independent replicates was used to determine statistical significance; **P < 0.01.

To explore whether Cdr1as exerts its cisplatin chemosensitivity effect by modulating APAF1 expression, we first constructed T24 and EJ cells cotransfected with the Cdr1as adenovirus and miR‐1270 mimics. In the cotransfected cells, increased APAF1 mRNA and protein expression levels induced by overexpressing Cdr1as were partly reversed (Fig. 7A). We then cotransfected the sh‐Cdr1as lentivirus and miR‐1270 inhibitor in T24 and EJ cells and observed the opposite phenomenon (Fig. 7B). Moreover, APAF1 expression in the same 32 pairs of bladder cancer tissues and adjacent normal tissues was measured, and correlation analysis revealed a moderate negative correlation between the expression of miR‐1270 and APAF1 (*R* = −0.6209, *P* < 0.001, Fig. 7C), as well as a moderate positive correlation between the expression of Cdr1as and APAF1 (*R* = 0.4098, *P* < 0.01; Fig. 7D). When it came back to xenografts, we found less miR‐1270 in Cdr1as OE tumors (Fig. 7E). Immunohistochemical staining and western blot also revealed that APAF1 levels were elevated in Cdr1as OE tumors (Fig. 7F,7G). Overall, the data demonstrate that Cdr1as plays a regulatory role through the Cdr1as/miR‐1270/APAF1 axis.

## 4 Discussion

In this study, we explored the effect of circRNA Cdr1as on the cisplatin chemosensitivity of bladder cancer and demonstrate the related regulatory mechanism, that is, the novel Cdr1as/miR‐1270/APAF1 axis. Our results indicate that upregulated Cdr1as could increase the cisplatin sensitivity of bladder cancer T24 and EJ cells accompanied by increased cell apoptosis and vice versa. Cdr1as can function as a molecular sponge of miR‐1270, which weakens the inhibitory effect of miRNA on the downstream target gene APAF1. Moreover, Cdr1as enhances the expression level of APAF1. Silencing APAF1 decreases cisplatin chemosensitivity and inactivates the apoptotic pathway. These findings demonstrate that Cdr1as dysregulation can contribute to the increased sensitivity of bladder cancer cells to cisplatin by cell apoptosis.

Cisplatin is one of the core drugs of many chemotherapeutic schemes, and resistance to this drug has drawn considerable research attention. Numerous bladder cancer patients are insensitive to cisplatin chemotherapy, which seriously restricts their long‐term survival (Godwin *et al.*, 2018). As a classic noncoding RNA, miRNA has been quite mature in chemoresistance. In our previous study, we found that miR‐218 could target Glut1, activate the oxidative stress pathway in bladder cancer, and enhance the sensitivity of bladder cancer cells to cisplatin chemotherapy (Li *et al.*, 2017). In recent years, the growing popularity of high‐throughput sequencing technology has enabled researchers to further investigate the expression and action mechanism of circRNA (Gao *et al.*, 2017), identified 18 differentially expressed circRNA in breast cancer by screening microarrays of drug‐resistant cell lines and sensitive cell lines, and then explored the potential chemoresistance mechanism of hsa_circ_0006528 (Kunpeng *et al.*, 2018). Cdr1as had been revealed to play important roles in esophageal cancer (Sang *et al.*, 2018), hepatocellular carcinoma (Xu *et al.*, 2017; Yu *et al.*, 2016), colorectal cancer (Tang *et al.*, 2017), and non‐small‐cell lung cancer (Zhang *et al.*, 2018). In our previous study, we found that Cdr1as was significantly downregulated in bladder cancer tissues and that Cdr1as could inhibit bladder cancer cell proliferation, migration, and invasion by sponging miRNA‐135a (Li *et al.*, 2018). In the present study, we found that Cdr1as overexpression in T24 and EJ cells could significantly reduce the survival rates of cells at different cisplatin concentrations. This finding suggests that Cdr1as enhances the sensitivity of bladder tumor cells to cisplatin and vice versa.

The molecular sponge mechanism of miRNA, as the classic regulatory pathway of circRNA, has been confirmed in many diseases, such as hepatocellular carcinoma (20), colorectal cancer (Tang *et al.*, 2017), and esophageal cancer (Sang *et al.*, 2018). In the present study, bioinformatics analysis, RNA pull‐down, and FISH experiments confirmed that Cdr1as could adsorb miR‐1270 in bladder cancer. Although miR‐1270 in tumors is rarely reported (Knox *et al.*, 2018; Zhong *et al.*, 2018), our study found that miR‐1270 expression is higher in tumor tissues than in adjacent normal tissues. We also discovered that upregulated miR‐1270 inhibits cell apoptosis and significantly reduces the cisplatin chemosensitivity of T24 and EJ cells. Therefore, Cdr1as may indirectly enhance the expression level of downstream target genes by adsorbing miR‐1270, thereby enhancing the sensitivity of cells to cisplatin chemotherapy.

Cell apoptosis is an important mechanism in the anticancer effect of cisplatin. Mitochondrial apoptosis is the endogenous pathway of apoptosis and the center of apoptosis regulation (Petrosillo *et al.*, 2003). During apoptosis, the BCL2 family is activated by apoptotic members (Park *et al.*, 2002). Then, BAX transfers to the mitochondria, and the formation of ion channels promotes the release of cytochrome C from the mitochondrial matrix to the cytoplasm (Park *et al.*, 2002). APAF1, which is the core molecule necessary to form ring‐like apoptosomes, forms a complex with cytochrome C, thereby activating the caspase‐9 precursor, which can activate caspase‐3 (Park *et al.*, 2002). Finally, the caspase cascade reaction is initiated to induce apoptosis (Park *et al.*, 2002). Previous studies have shown that APAF1 overexpression in cisplatin‐resistant Hela cell lines can partly reverse cisplatin chemoresistance, thus suggesting that APAF1 is involved in the regulation of cisplatin resistance (Kamarajan *et al.*, 2001). In the present study, we detected apoptotic proteins in T24 and EJ cells overexpressing Cdr1as and found significantly increased APAF1 expression. Bioinformatics software and experimental results confirm that APAF1 is the direct target of miR‐1270. The cell apoptosis rates and cisplatin chemosensitivity of bladder cancer T24 and EJ cells were also significantly increased by knocking down APAF1.

## 5 Conclusions

Based on our previous study and the present findings, we conclude that Cdr1as can adsorb miR‐1270 in bladder cancer, restore its suppressed APAF1 expression level, induce apoptosis, and increase cisplatin chemosensitivity. Targeting the Cdr1as/miR‐1270/APAF1 axis presents a new strategy to enhance the cisplatin chemosensitivity of bladder cancer patients. Cdr1as is potentially playing role in predicting and monitoring cisplatin chemosensitivity in patients with bladder cancer.

## Conflict of interest

The authors declare no conflict of interest.

## Author contributions

QL and HY conceived, designed, and analyzed experiments; WY, RZ, JW, JH, and XY performed experiments and wrote the manuscript; HY, HL, XZ, PL, JT, JW, and MG helped in conceiving and/or analyzing the experiments and provided reagents.

## Supporting information


**Fig. S1**. Relative abundance of Cdr1as using two different normalizers. (A) Relative abundance of Cdr1as by qRT‐PCR using β‐Actin as a normalizer in all available bladder cancer cell lines. (B) Relative abundance of Cdr1as by qRT‐PCR using GAPDH as a normalizer in all available bladder cancer cell lines. (C) Relative abundance of Cdr1as by qRT‐PCR using GAPDH as a normalizer in T24 and EJ affected with Cdr1as or GFP. (D) The expression levels of Cdr1as were performed by qRT‐PCR using GAPDH as a normalizer in bladder cancer cells transfected with three kinds of sh‐Cdr1as or sh‐CTL (control) vector respectively. Data represent the mean ± SD from three independent experiments. Student’s t‐test with two biological independent replicates were used to determine statistical significance; *P < 0.05, **P < 0.01

**Fig. S2**. Knocking down Cdr1as could reduce the cisplatin chemosensitivity in TCCSUP and 5637 cell lines. (A) Relative abundance of Cdr1as in TCCSUP and 5637 cell lines infected by sh‐Cdr1as lentivirus by qRT‐PCR. (B, C) Cells viability was determined by the CCK‐8 method to be promoted by sh‐Cdr1as‐transfected cells in a series of dilute concentrations of cisplatin. (D) Knocking down Cdr1as could reduce the sensitivity of TCCSUP and 5637 cells to cisplatin. Data represent the mean ± SD from three independent experiments. Student’s t‐test with two biological independent replicates were used to determine statistical significance; *P < 0.05, **P < 0.01.

**Fig. S3**. The nude mice metastasis models. (A) Almost no signal detected in a normal nude mouse (Scale bar, 10mm) by IVIS 100 Imaging System and its lungs (Scale bar, 5mm). (B) High signal detected in a mouse with metastasis (Scale bar, 10mm) by IVIS 100 Imaging System and its lungs (Scale bar, 5mm).

**Fig. S4**. Knockdown of Cdr1as could decrease cell apoptosis in TCCSUP and 5637 cells. (A, B) Cell apoptosis analyzed by flow cytometry in TCCSUP and 5637 cells after the knockdown of Cdr1as. Data represent the mean ± SD from three independent experiments. Student’s *t*‐test with two biological independent replicates were used to determine statistical significance; *P < 0.05, **P < 0.01

**Fig. S5**. MiR‐7, miR‐135a and miR‐1246 showed no influence on the sensitivity to cisplatin in T24 cells. (A‐C) Cells viability was determined by the CCK‐8 method to be of no significant differences among miR‐7, miR‐135a and miR‐1246 over‐expressed cells in a series of dilute concentrations of cisplatin. (D) Over‐expression of miR‐7, miR‐135a or miR‐1246 could not affect the IC50 of T24 cells to cisplatin. Data represent the mean ± SD from three independent experiments. Student’s t‐test with two biological independent replicates were used to determine statistical significance; *P < 0.05, **P < 0.01

**Fig. S6**. Representative IHC analysis of APAF1 protein in bladder cancer tissues. Magnification: 200 × (top) and 400× (bottom) (Scale bar, 10μm).

**Fig. S7**. Kaplan‐Meier survival analysis for OS of patients in an aggregate bladder cancer dataset according to APAF1 and miR‐1270 expression status. The *P* value was determined using the log‐rank test. Original data was obtained from TCGA.

**Table S1**. Demographic and clinical features of 32 patients with bladder cancer.

**Table S2**. List of all potential targets of miR‐1270 predicted by 4 databases.

**Table S3**. List of 189 common potential targets of miR‐1270 predicted by 4 databases.
