# Supplementary figures and images for: Circular RNA Cdr1as sensitizes bladder cancer to cisplatin by upregulating APAF1 expression through miR‐1270 inhibition
Source: Mol Oncol. 2019 Jun 9;13(7):1559–76. doi: 10.1002/1878-0261.12523 (PMC6599840; doi:10.1002/1878-0261.12523)

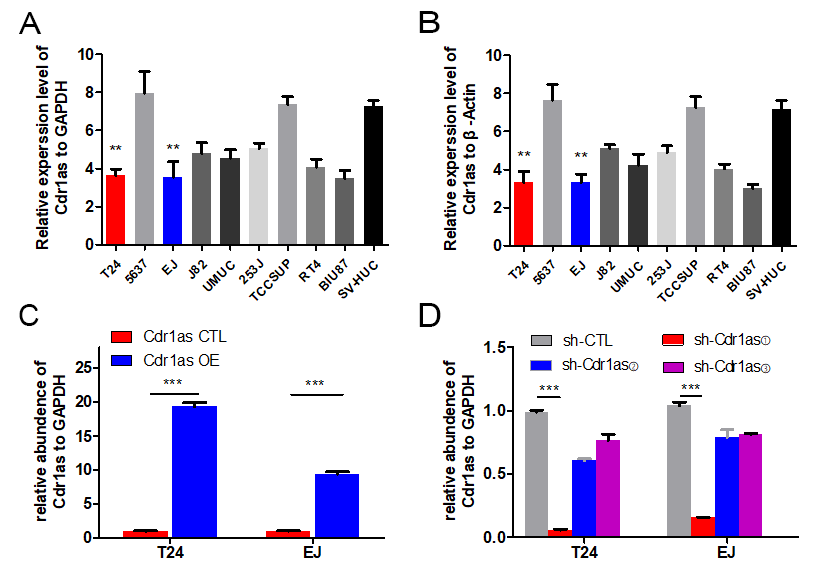

Supplement: Supplementary file 1 — Fig. S1. Relative abundance of Cdr1as using two different normalizers. (A) Relative abundance of Cdr1as by qRT‐PCR using β‐Actin as a normalizer in all available bladder cancer cell lines. (B) Relative abundance of Cdr1as by qRT‐PCR using GAPDH as a normalizer in all available bladder cancer cell lines. (C) Relative abundance of Cdr1as by qRT‐PCR using GAPDH as a normalizer in T24 and EJ affected with Cdr1as or GFP. (D) The expression levels of Cdr1as were performed by qRT‐PCR using GAPDH as a normalizer in bladder cancer cells transfected with three kinds of sh‐Cdr1as or sh‐CTL (control) vector respectively. Data represent the mean ± SD from three independent experiments. Student’s t‐test with two biological independent replicates were used to determine statistical significance; *P < 0.05, **P < 0.01 [file MOL2-13-1559-s001.tif]

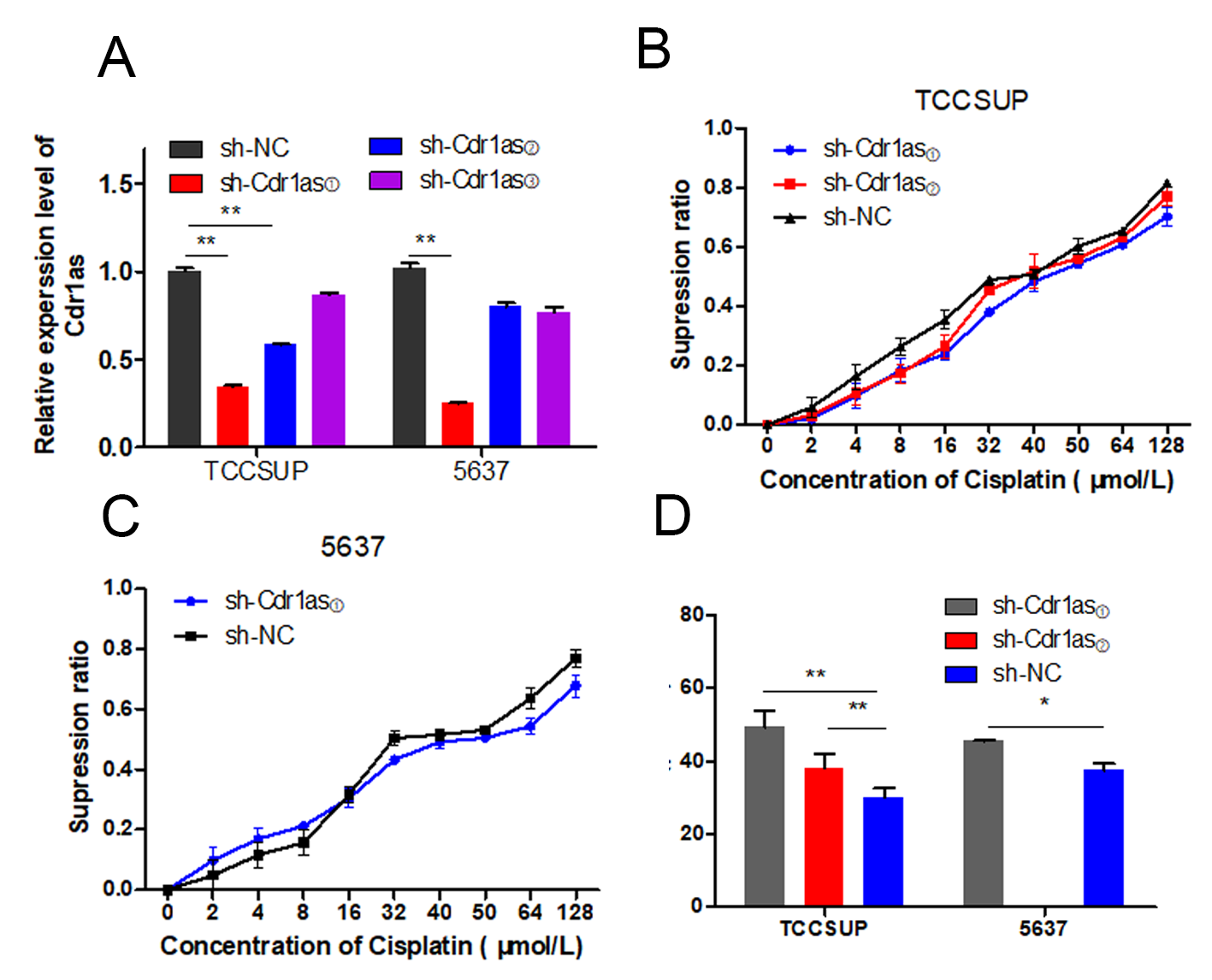

Supplement: Supplementary file 2 — Fig. S2. Knocking down Cdr1as could reduce the cisplatin chemosensitivity in TCCSUP and 5637 cell lines. (A) Relative abundance of Cdr1as in TCCSUP and 5637 cell lines infected by sh‐Cdr1as lentivirus by qRT‐PCR. (B, C) Cells viability was determined by the CCK‐8 method to be promoted by sh‐Cdr1as‐transfected cells in a series of dilute concentrations of cisplatin. (D) Knocking down Cdr1as could reduce the sensitivity of TCCSUP and 5637 cells to cisplatin. Data represent the mean ± SD from three independent experiments. Student’s t‐test with two biological independent replicates were used to determine statistical significance; *P < 0.05, **P < 0.01. [file MOL2-13-1559-s002.tif]

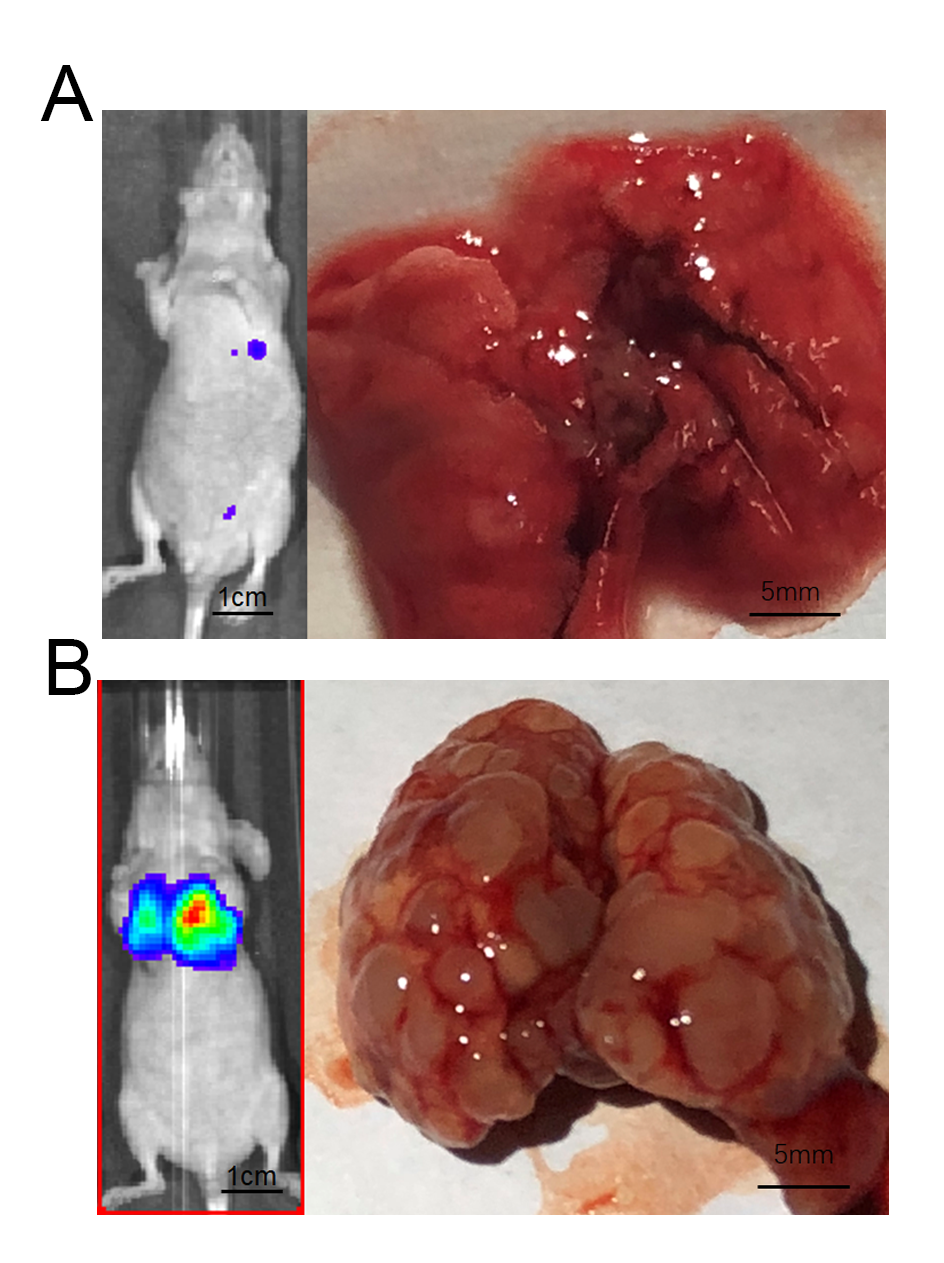

Supplement: Supplementary file 3 — Fig. S3. The nude mice metastasis models. (A) Almost no signal detected in a normal nude mouse (Scale bar, 10mm) by IVIS 100 Imaging System and its lungs (Scale bar, 5mm). (B) High signal detected in a mouse with metastasis (Scale bar, 10mm) by IVIS 100 Imaging System and its lungs (Scale bar, 5mm). [file MOL2-13-1559-s003.tif]

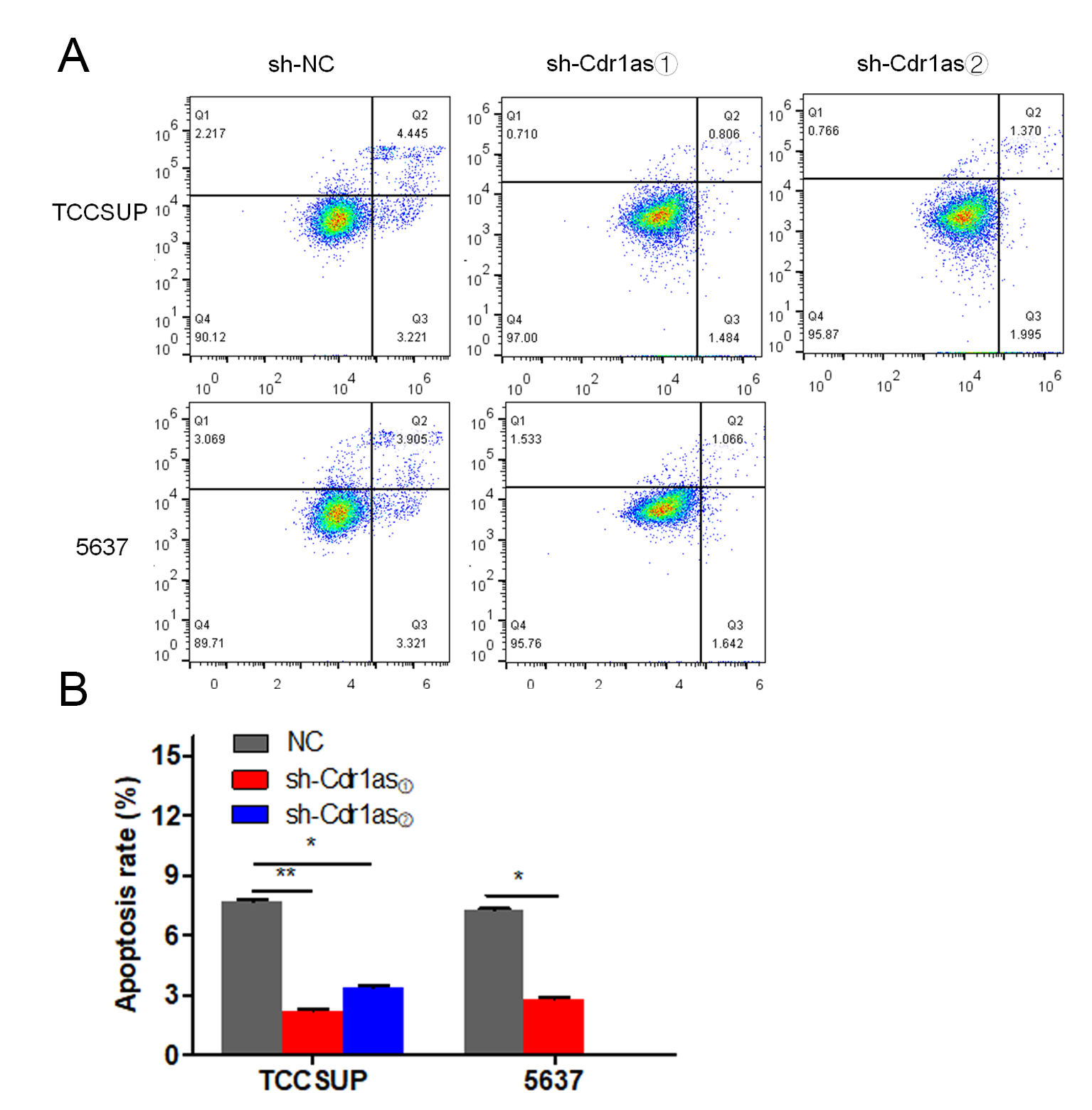

Supplement: Supplementary file 4 — Fig. S4. Knockdown of Cdr1as could decrease cell apoptosis in TCCSUP and 5637 cells. (A, B) Cell apoptosis analyzed by flow cytometry in TCCSUP and 5637 cells after the knockdown of Cdr1as. Data represent the mean ± SD from three independent experiments. Student’s t‐test with two biological independent replicates were used to determine statistical significance; *P < 0.05, **P < 0.01 [file MOL2-13-1559-s004.tif]

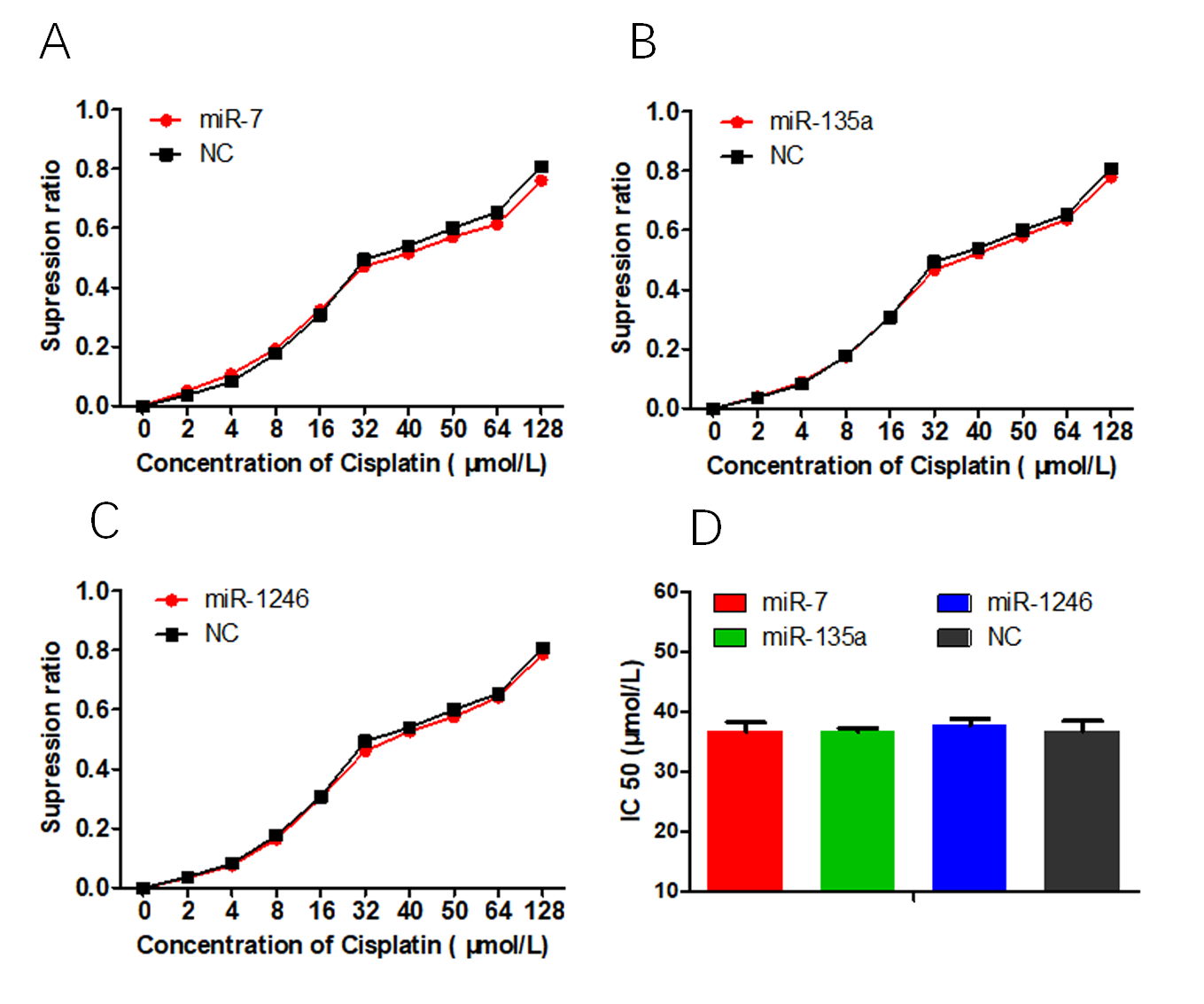

Supplement: Supplementary file 5 — Fig. S5. MiR‐7, miR‐135a and miR‐1246 showed no influence on the sensitivity to cisplatin in T24 cells. (A‐C) Cells viability was determined by the CCK‐8 method to be of no significant differences among miR‐7, miR‐135a and miR‐1246 over‐expressed cells in a series of dilute concentrations of cisplatin. (D) Over‐expression of miR‐7, miR‐135a or miR‐1246 could not affect the IC50 of T24 cells to cisplatin. Data represent the mean ± SD from three independent experiments. Student’s t‐test with two biological independent replicates were used to determine statistical significance; *P < 0.05, **P < 0.01 [file MOL2-13-1559-s005.tif]

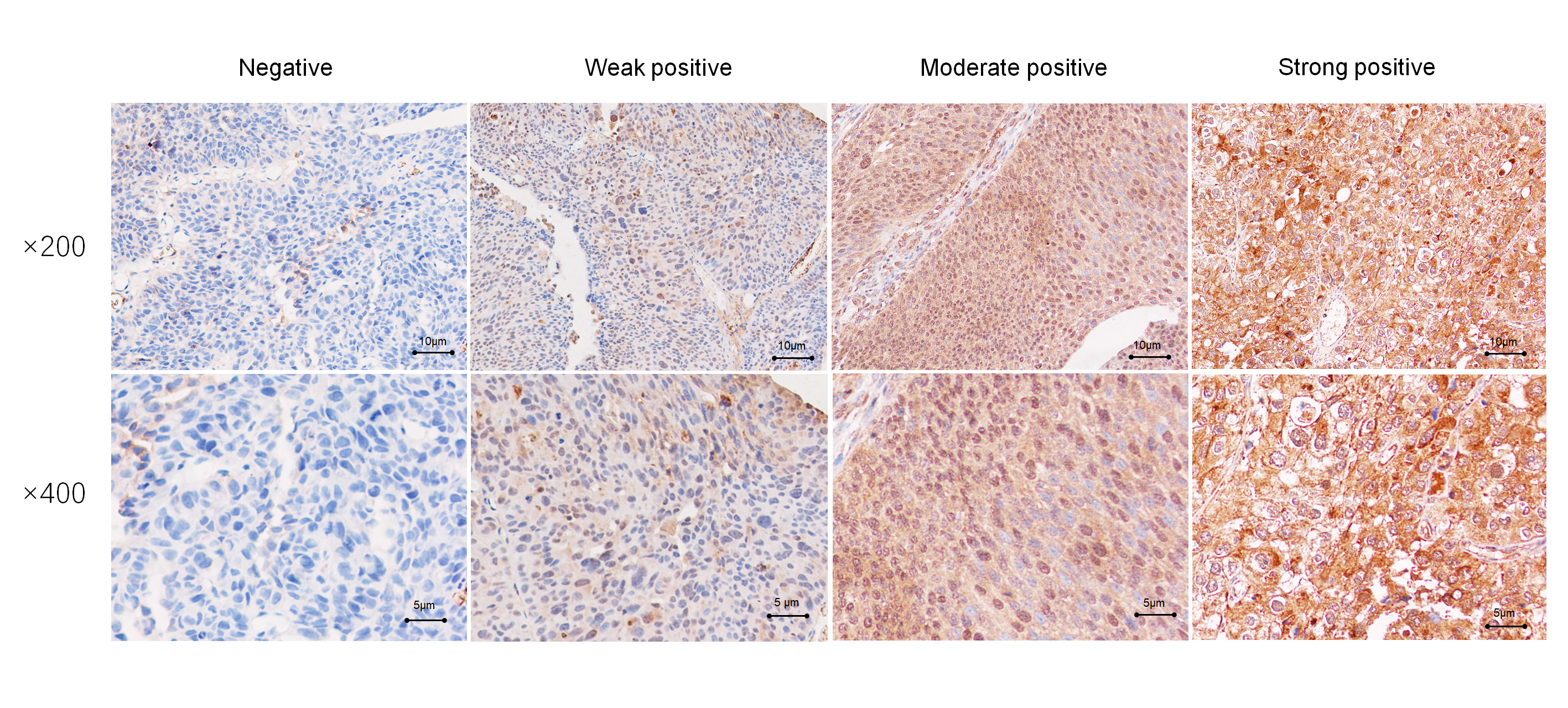

Supplement: Supplementary file 6 — Fig. S6. Representative IHC analysis of APAF1 protein in bladder cancer tissues. Magnification: 200 × (top) and 400× (bottom) (Scale bar, 10μm). [file MOL2-13-1559-s006.tif]

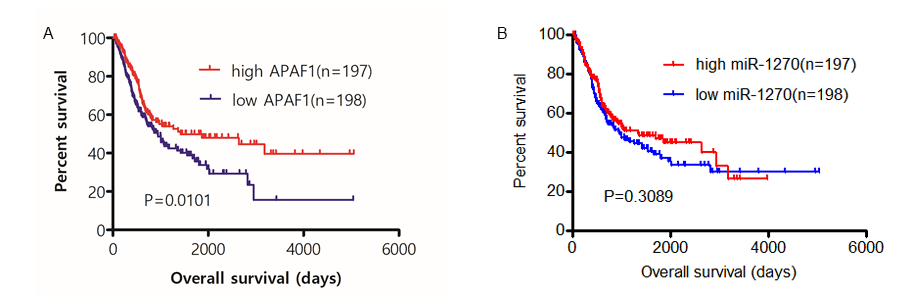

Supplement: Supplementary file 7 — Fig. S7. Kaplan‐Meier survival analysis for OS of patients in an aggregate bladder cancer dataset according to APAF1 and miR‐1270 expression status. The P value was determined using the log‐rank test. Original data was obtained from TCGA. [file MOL2-13-1559-s007.tif]
