# Supplementary material for: Circular RNA Cdr1as sensitizes bladder cancer to cisplatin by upregulating APAF1 expression through miR‐1270 inhibition
Source: Mol Oncol. 2019 Jun 9;13(7):1559–76. doi: 10.1002/1878-0261.12523 (PMC6599840; doi:10.1002/1878-0261.12523)
Supplement: Supplementary file 8 — Table S1. Demographic and clinical features of 32 patients with bladder cancer. [file MOL2-13-1559-s008.docx]

**Table S1** Demographic and clinical features of 32 patients with bladder cancer

| Variable | N | % |
| --- | --- | --- |
| Cases | 32 | 100 |
| Age(years) |  |  |
| >=68 | 19 | 59.4 |
| <68 | 13 | 40.6 |
| Gender |  |  |
| Male | 32 | 100 |
| Female | 0 | 0 |
| Tobacco smoking status | |  |
| Never | 23 | 71.9 |
| Positive | 9 | 28.1 |
| Alcohol consumption status | |  |
| Never | 22 | 68.8 |
| Positive | 10 | 31.2 |
| Tumor stage | |  |
| I | 8 | 25 |
| II | 17 | 53.1 |
| III | 3 | 9.4 |
| IV | 4 | 12.5 |
| Tumor grade | |  |
| PUNLMP | 2 | 6.3 |
| Low grade | 5 | 15.6 |
| High grade | 25 | 78.1 |
